# Microvesicles as Vehicles for Tissue Regeneration: Changing of the Guards

**DOI:** 10.1007/s40139-016-0115-5

**Published:** 2016-08-27

**Authors:** Nikolaos Panagiotou, R. Wayne Davies, Colin Selman, Paul G. Shiels

**Affiliations:** 1 Wolfson Wohl, Translational Research Centre, Institute of Cancer Sciences, MVLS, University of Glasgow, Garscube Estate, Switchback Road, Glasgow, G61 1QH UK; 2 School of Informatics, Institute of Neural and Adaptive Computation, Informatics Forum, University of Edinburgh, 10 Crichton Street, Edinburgh, EH8 9AB UK; 3 Graham Kerr, Institute of Biodiversity Animal Health and Comparative Medicine, University of Glasgow, Glasgow, G12 8QQ UK

**Keywords:** Microvesicles, extracellular vesicles, Stem cells, Tissue regeneration, Regenerative medicine, Ageing

## Abstract

**Purpose of Review:**

Microvesicles (MVs) have been recognised as mediators of stem cell function, enabling and guiding their regenerative effects.

**Recent Findings:**

MVs constitute one unique size class of extracellular vesicles (EVs) directly shed from the cell plasma membrane. They facilitate cell-to-cell communication via intercellular transfer of proteins, mRNA and microRNA (miRNA). MVs derived from stem cells, or stem cell regulatory cell types, have proven roles in tissue regeneration and repair processes. Their role in the maintenance of healthy tissue function throughout the life course and thus in age related health span remains to be elucidated.

**Summary:**

Understanding the biogenesis and mechanisms of action of MVs may enable the development of cell-free therapeutics capable of assisting in tissue maintenance and repair for a variety of age-related degenerative diseases. This review critically evaluates recent work published in this area and highlights important new findings demonstrating the use of MVs in tissue regeneration.

## Introduction

A large component of physiological homeostasis has been attributed to the action of extracellular vesicles (EVs), which are thought to have a key role in tissue maintenance and repair. EVs can be defined as all membranous vesicles that are secreted by cells and that encapsulate bioactive molecules, including a variety of proteins and nucleic acids. EVs consist of at least three distinct vesicle groups characterised by size, shape and point of cellular origin. These comprise Microvesicles (MVs), exosomes and apoptotic bodies. MVs vary in size, ranging from 100 to 1000 nm in diameter [1, 2], while exosomes and apoptotic bodies have a diameter less than 100 nm [1–5]. While MVs and exosomes are the focus of the majority of biomedical research, the functions and character of other EVs are less well understood. EVs can be found in most human biological fluids and interestingly, have been reported to contain non-coding RNAs [6–8] and double-stranded DNA [9]. It has been postulated that EVs provide a means of intercellular communication both in physiological and pathological states, by transferring their molecular payloads from donor to recipient cells throughout the human body [8, 10]. This transfer of biologically active molecules can generate functional changes in the recipient cells [6] and may also provide a molecular basis for the spread of allostatic load and the effects of non-cell autonomous senescence across the body [11].

A direct sequitur of this hypothesis is that both the MV class and the exosome class of EVs have been experimentally shown to significantly assist and improve the rate of paracrine-mediated tissue damage repair. Specifically, as part of the repair process, the phenotype of the recipient cells can be altered by three proposed mechanisms through interactions with MV-associated components. Firstly, by translation of the mRNA molecules present in MVs, once endocytosis has taken place and the mRNAs are located within the recipient cell cytoplasm. Secondly, by negative regulation of the recipient cell mRNA molecules by the MV-associated microRNA (miRNA) regulators. Thirdly, by direct MV-associated protein activity within the recipient cell. These will now be discussed with a primary focus on the contribution of MVs to tissue and organ regeneration and in comparison to Exosomes.

## Microvesicles

MVs were once thought to be cell debris, but recent research has indicated that they have a proven role in intercellular communication, stem cell regulation and as potential therapeutic entities for tissue repair [10, 12, 13]. They are the product of budding from the cell membrane and they are shed from almost all cell types as free spherical vesicles [14]. Consequently, MVs are found in most biological fluids, such as plasma, urine, synovial fluid and bronchial lavage fluid [15, 16]. Cells are triggered to generate high quantities of MVs in response to a variety of stimuli, such as differentiation, senescence and stress; however, the mechanisms are not clear [17]. Furthermore, increased intracellular calcium levels are suggested to enhance MV production, via phospholipid position changing and flipping of phosphatidyl-serine from the inside to the outside of the cell membrane [15, 17, 18]. Here, it needs to be highlighted that MVs are different from and should not be confused with exosomes, which are smaller than MVs in size (40–100 nm in diameter) [4] and are secreted by cells via a completely different mechanism that involves fusion of multivesicular endosomes with the plasma membrane [15, 19, 20]. MVs must also be distinguished from apoptotic bodies (1000–4000 nm in diameter) that are secreted from dying cells during apoptosis [6].

## Mode of Action

MVs have been recognized as carriers of protein and genetic material, such as mRNA, miRNA and other small non-coding RNAs, that can be secreted from almost all types of cells and affect the phenotype of recipient cells [21], either transcriptionally or post-transcriptionally [7]. Interestingly, both MVs and exosomes contain and transfer a particular set of mRNA and miRNA molecules, some of which are not present in the cytoplasm of the donor cell, suggesting that these molecules may have been produced by the donor cell in order to facilitate intercellular communication and affect the phenotype and processes of recipient cells [8].

MVs derived from stem cells, or stem cell regulatory cells, have been consistently shown to play an essential role in stem cell-mediated repair of tissue damage. While different types of stem cells have been used in various cell transplantation therapies, no evidence currently exists to suggest that any significant number of the adult-derived stem cells have differentiated and subsequently produced large populations of organ-specific cells in vivo. Additionally, it is apparent that the transplantation and subsequent action of different stem cell types under specific tissue damage produces similar outcomes with administration of conditioned medium from mesenchymal stem cells (MSCs) in terms of tissue regeneration [22]. Hence, these observations, along with findings that demonstrate the secretion of MV paracrine elements from stem cells and the presence of MVs in stem cell-conditioned medium, indicate that stem cells might exert their regenerative properties through MV-mediated paracrine mechanisms [22, 23].

Recently, the miRNA signatures for the aforementioned intercellular communication factors from stem cells and stem cell regulatory cell types have been investigated as potential therapeutic agents [24]. Extracellular miRNAs can be found in MVs in the bloodstream as well as in other body fluids, and they can be transferred horizontally between cells [25]. miRNAs typically comprise 22 nucleotide, non-coding RNA molecules that act as negative regulators of gene expression by suppressing the expression of genes post-transcriptionally [26]. The donor cell miRNA molecules bind to complementary sequences in the coding or 3′ untranslated region of the recipient cell mRNAs and inhibit their translation or promote their degradation. The donor cell miRNA molecules bind to complementary sequences in the coding or 3′ untranslated region of the recipient cell mRNAs and inhibit their translation or promote their degradation. In this way, miRNAs can silence the translation of mRNA and subsequently influence a range of biochemical processes, including proliferation, apoptosis and regulation of metabolism. Notably, miRNAs may also function to regulate cellular differentiation processes and tissue homeostasis. For instance, terminal differentiation of osteoclasts and angiogenesis, which is an essential process for organ growth and repair [27], are regulated by miRNAs [28]. Furthermore, it has been shown that stem cell MVs alone can promote angiogenesis in vivo in a similar fashion to stem cell treatment [29]. Moreover, MVs isolated from embryonic stem cells have been shown to induce reprogramming of hematopoietic progenitor cells [30]. Thus, MVs are excellent candidates for novel therapeutic tools for tissue regeneration, through angiogenesis, stem cell differentiation, cell migration and activation of anti-apoptosis.

Ageing is also believed to be regulated, in part, by miRNAs, either circulating free or encapsulated in EVs, such as MVs and exosomes, raising the possibility that age-related paracrine signalling mechanisms employed by stem cells may be implicated in processes tightly associated with age-associated tissue degeneration [26, 31, 32]. Different expression levels of several miRNAs have been found with age in a variety of species, from human to worm, where upregulation of specific miRNAs induces cell senescence with ageing. Moreover, miRNAs enclosed in MVs and exosomes have been shown to play a role in a number of age-related diseases, such as atherosclerosis, osteoporosis, Alzheimer’s disease and type 2 diabetes mellitus [33]. Consequently, paracrine signalling mechanisms may be implicated in processes tightly associated with age related tissue maintenance and damage repair. Consequently, MVs also have the potential to become a cell-free therapeutic agent for treating age-related degenerative diseases, as well as for achieving an increase in healthy lifespan through the mitigation of the drivers of age-related loss of physiological function. This approach also has the important additional bonus of providing a therapeutic vehicle that avoids the ethical complications and technical hurdles that are still associated with stem cell transplantation therapies [34, 35]. However, scientific breakthroughs in this area of research are essential so as to produce more data and advance our knowledge in order to make MV therapies a reality.

The above-mentioned observations have added a new dimension in the research on MVs, stem cells and regenerative medicine. Hence, MVs may provide a novel means for the development of prospective strategies of cell-free regenerative therapies ultimately aiming to tackle otherwise intractable pathologies, improve age-related health span and mitigate the effects of degenerative diseases by tissue regeneration [36].

The first step towards exemplifying the use of MVs as a cell-free therapeutic for the treatment of tissue and organ damage have now begun [24, 37]. One recent exemplification has been the use of rat MVs to regenerate damaged pancreata in mice.

## Pancreas Recovery and Protection Against Kidney Damage

Pathfinder cells (PCs) are a putative stem cell regulatory cell type that has been demonstrated to enable tissue regeneration in two models of solid organ damage (kidneys and pancreas), working across both concordant and discordant xenotransplantation barriers in vivo. PCs have demonstrated the ability to stimulate regeneration following acute ischaemic renal damage [38] and to completely reverse the effects of streptozotocin (SZT)-induced diabetes in mice [39]. In both instances, the mode of action of the PCs, independently of the species of origin, was paracrine in nature, suggesting that PC-derived MVs and their cargo could play a key role in regulating organ repair.

A recent study by McGuinness et al. has elegantly demonstrated that MVs derived from rat PCs could stimulate functional recovery of pancreata in mice with SZT-induced diabetes, exactly as PCs themselves. In addition, this study highlighted that only the PC MV class and not PC exosomes could stimulate functional recovery of the pancreas in vivo [40•]. This, to the best of our knowledge, is the first time that a direct functional comparison has been made between the regenerative capacity of MVs and exosomes in an in vivo model. This observation suggests that MVs from other cell types may also be superior to exosomes in enabling tissue regeneration in vivo.

MVs derived from adult human MSCs have also been demonstrated to be protective against renal damage following glycerol-induced, ischemia–reperfusion and cisplatin-induced acute kidney injury via horizontal transfer of mRNA and miRNA molecules [41–43]. Several other studies have also indicated that MSC MVs can reverse acute and chronic kidney injury in a variety of experimental models, via inhibition of apoptosis and stimulation of proliferation [44, 45].

However, MSC exosomes have not been shown to be capable of assisting tissue regeneration and inducing positive effects in in vivo models of pancreatic and/or renal damage. Interestingly though, it is worth highlighting that MSC exosomes have been previously observed to assist wound healing in vitro [46]. In other words, it appears that the regenerative effect of exosomes, either of MSC or PC origin, is limited to in vitro models as far as pancreas and kidney damage are concerned. Hence, the factors underlying variation in the regenerative efficacy of exosomes between in vitro and in vivo pancreatic and renal studies are worth addressing in future research. Thus, it becomes apparent that it is vitally important to both explore and recognise the differences between these two types of EVs. Understanding the differences between MVs and exosomes in processes such as biogenesis and interaction with recipient cells, as well as mode of action, both in vitro and in vivo, could prove to be vital in devising therapeutic strategies with highly potential outcomes in the near future.

Finally, endothelial progenitor cells have been used as a source of MVs with potential therapeutic and protective properties. These MVs have been shown to protect against kidney damage following ischemia–reperfusion in rats, through a mechanism involving miRNA delivery to resident renal cells [47]. Nevertheless, it is clear that more studies are needed in order to fully assess the efficacy and complete mode of action of MVs in pancreas and renal repair before their translation into human therapies [48].

## Cardiac Regeneration

MVs have also been suggested as potential therapeutic effectors in cardiac regeneration. For the first time, MVs derived from human-induced pluripotent stem cells (iPSCs) were shown to transmit proteins and RNAs to cardiac MSCs in vitro, affecting their transcriptome and proteome and enhancing their cardiac and endothelial differentiation potential [49•]. Paracrine factors derived from these iPSCs were later isolated and used in an in vivo setting in which mice were exposed to acute myocardial ischemia/reperfusion injury. The results suggested that both the MVs and exosomes from the iPSCs were able to transfer miRNAs to the ischemic myocardium and protect the cardiomyocytes through the inhibition of apoptosis [50]. Moreover, a study by Feng et al. showed that the MSC paracrine factors are involved in the protection of ischemic cardiomyocytes by transmission of a specific miRNA, miR-22, leading to reduced apoptosis. This observation was validated in MSCs and neonatal cardiomyocytes co-cultures in vitro, and in vivo using a myocardial infarction mouse model [51]. While more future in vivo studies are warranted in order to prove this concept, these findings described above open the way for the potential development of safe acellular therapeutic applications, avoiding the challenges, both ethical and technical, of pluripotent stem cell transplantation therapies [35], not only for cardiac regeneration but also for the repair of other tissues.

## Retinal Regeneration

The therapeutic potential of MVs has also been investigated in the context of retinal regeneration. MVs were isolated from mouse embryonic stem cells, and their capability to affect Müller cells (a type of quiescent retinal glial cell) and alter them into a more permissive state for regeneration of damaged retina was investigated. It was subsequently shown that MVs were capable of transferring mRNAs and miRNAs to cultured Müller cells, enabling them to re-enter the cell cycle by induction of pluripotency. This was then followed by differentiation into cells of retinal lineage [52•]. The same group has on-going studies testing the therapeutic effect of MVs in vivo, in mice with damaged retinas, and the outcomes would be of great interest for establishing novel applications of MVs in tissue regeneration.

## Treatment of Lung Injury

MSCs have previously been discussed as an attractive therapeutic approach for the treatment of acute lung injury (ALI) and acute respiratory distress syndrome (ARDS). In addition, emphasis has been given to the potential role of the paracrine factors that these cells secrete [53, 54]. Similarly, MSCs have been used as a therapeutic approach for combating developmental lung injury and lung vascular diseases [55]. The significance of paracrine factors in the treatment of the above disorders was later highlighted in vitro by restoring sodium transport and preserving epithelial permeability in an ALI model of rat alveolar epithelial cells, after addition of MSC-conditioned media, which included EV paracrine factors, such as MVs and exosomes [56]. Previously, exosomes derived from bone marrow MSCs have been shown to provide a protective effect during hypoxia-induced pulmonary hypertension. This protective effect was mitigated by suppression of hyper-proliferative pathways, such as the STAT3-mediated signalling pathway, induced by hypoxia [57]. More recently, MVs have been shown to have a significant therapeutic potential in treating ALI and ARDS in vivo, in mice that had endotoxin-induced ALI with *Escherichia coli* infection. The MVs that were used for this purpose were derived from human MSCs of bone marrow origin. These MVs were shown to have a positive therapeutic effect and mitigate the effects of ALI in mice [58].

## Nervous System Repair Therapies

MVs have also been discussed as critical to a variety of events in the nervous system. Interestingly, they could both provide protection from neurodegeneration and have a central role in the propagation of neurotoxicity [59]. Exosomes have been implicated in spreading the key disease molecules of Parkinson’s and Alzheimer’s diseases through the brain [60], and MVs may also play a part in this important disease process. As far as their protective action is concerned, MSC-based therapies applied in numerous models of neurodegenerative disorders have provided strong evidence of paracrine regeneration effects [61]. Xin et al. demonstrated that exosome paracrine factors originating from MSCs can have a positive effect on neural cells after experimentally induced stroke in rodents, by the transfer of the miR-133b miRNA [62–64]. More recently, another group showed both in vitro and in vivo that MVs isolated from macrophages, which have been highly associated with regeneration of peripheral nerves, stimulated proliferation and migration of Schwann cells [65•]. Schwann cells are cells of the peripheral nervous system that contribute to axonal regeneration after nerve injury. These results provide a novel strategy for the development of nervous system repair therapeutics with the use of either MVs or exosomes. Nevertheless, the potential of EVs to transfer toxic molecules, as may occur in nervous system diseases, should be taken into consideration and thoroughly investigated, before safe and efficient therapies can be devised for the repair of nerve damage.

## Conclusions

Overall, robust evidence exists to support the premise that MVs are capable of horizontally transferring proteins, mRNA and miRNA from donor to recipient cells and subsequently affecting their phenotype [6, 8, 30]. MVs can stimulate functional changes in the recipient cells by three proposed mechanisms; protein action, mRNA translation and negative post-transcriptional regulation of gene expression facilitated by miRNA action. These phenotypic changes conferred by MVs through paracrine mechanisms allow for stem cell regulation and therapeutic efficacy during tissue regeneration via anti-apoptosis and enhanced angiogenesis.

The potential of MVs to develop effective therapeutic interventions in the near future has been demonstrated numerous times over the past few years, and their role in tissue regeneration through the horizontal transfer of bioactive molecules in intercellular communication has become widely accepted. Additionally, genetic engineering may be used to modify MV-producing cells to give rise to higher quantities of MVs with enhanced tissue regeneration efficiency in vivo, based on the presence and possibly quantity of their paracrine effectors, such as miRNAs. Alternatively, production of synthetic MVs that carry the required bioactive apparatus necessary to facilitate the transfer of damage repair signals could be deployed, for the treatment of tissue impairment in degenerative diseases or tissue decay that comes with ageing.

MVs are in clinical trials and/or moving into clinical trials on a number of fronts [66]. However, for safe and successful clinical applications to be developed, more research is essential into the field of MVs, so as to further understand their biogenesis, transport and biological mode of action. It is also important to identify all the bioactive species that are found within MVs and understand how they change depending on the donor cells and what their exact mechanism of action is once they contact the recipient cells. Moreover, it would be of significant interest to understand how the production of MVs, their interaction with target cells and/or their payload changes with age. In other words, it is important that MVs remain therapeutically efficient and safe for use in tissue regeneration with ageing, as ageing is a major risk factor for many degenerative diseases, apart from the tissue decay that is its physiological outcome. Also, it is worth exploring the possibility that MVs and other EVs, such as exosomes, act as vehicles for spreading age-related allostatic load within the body. Additionally, it is of vital importance to become able to exploit their regenerative and anti-apoptotic properties more accurately and efficiently for different organs, tissues and cell types. In order to do so, it is important to develop large scale MV production, as well as isolation and purification methods. Also it is essential that technologies are developed that are capable of efficiently separating MVs from apoptotic bodies and investigate what cell populations and which cargoes are suitable for devising different therapeutic strategies. Finally, it is necessary to provide guidance on various important ethical, technical, legal and regulatory issues that could potentially arise, concerning the use of MVs as a therapeutic intervention for tissue damage, degenerative diseases and ageing.
